# Depth-resolved carbon dioxide and methane concentrations in 522 lakes, ponds, and reservoirs worldwide

**DOI:** 10.1038/s41597-026-06751-0

**Published:** 2026-02-19

**Authors:** Joseph S. Rabaey, Abigail S. L. Lewis, Katrin Attermeyer, Patrick Aurich, Sheel Bansal, Maciej Bartosiewicz, Brittni L. Bertolet, Ingeborg Bussmann, Sarah B. Cadieux, Elisa Calamita, Camilla Capelli, Cayelan C. Carey, Carmen Cillero, Francois Clayer, Sofia L. D’Ambrosio, Thomas A. Davidson, Bridget R. Deemer, Blaize A. Denfeld, Werner Eckert, Chiara Esposito, Phillip Ford, Adrianna Gorsky, Natalie A. Griffiths, Hans-Peter F. Grossart, David P. Hamilton, Meredith A. Holgerson, Brian J. Huser, Tomoya Iwata, Joachim Jansen, Stuart E. Jones, Sari Juutinen, Pirkko Kortelainen, Matthias Koschorreck, Theis Kragh, Alo Laas, Tuula Larmola, Saskia Läubli, Isabelle Laurion, Moritz F. Lehmann, Liu Liu, Pertti J. Martikainen, Anna Matoušů, Stephen A. McCord, Jorge J. Montes-Pérez, Daniele Nizzoli, César Ordóñez, Mike Peacock, Rachel M. Pilla, Vilmantas Prėskienis, Junbing Pu, Tenna Riis, Taija Saarela, Arianto B. Santoso, Carsten J. Schubert, Armando Sepulveda-Jauregui, Bradford S. Sherman, Jonas S. Sø, Katherine J. Stenehjem, Kristin E. D. Strock, Kenji Tsuchiya, Katrin Wendt-Potthoff, Gesa A. Weyhenmeyer, Petr Znachor, Jakob Zopfi

**Affiliations:** 1 Large Lakes Observatory, University of Minnesota-Duluth, Duluth, MN USA; 2 Smithsonian Environmental Research Center, Edgewater, MD 21037 USA; 3 WasserCluster Lunz - Biologische Station, Lunz am See, Austria; 4 Department of Functional and Evolutionary Ecology, University of Vienna, Vienna, Austria; 5 Helmholtz Centre for Environmental Research – UFZ, Leipzig, Germany; 6 U.S. Geological Survey, Northern Prairie Wildlife Research Center, Jamestown, USA; 7 Institute of Geophysics, Polish Academy of Science, Warsaw, Poland; 8 Department of Earth and Environmental Sciences, Rutgers University Newark, Newark, USA; 9 Alfred-Wegener-Institut, Helmholtz Zentrum für Polar- und Meeresforschung, Department of Shelf Sea System Ecology, Helgoland, Germany; 10 Department of Earth and Environmental Sciences, Rensselear Polytechnic Institute, Troy, USA; 11 Department of Geosciences, Eberhard Karls University of Tübingen, 72076 Tübingen, Germany; 12 University of Applied Sciences and Arts of Southern Switzerland, Mendrisio, Switzerland; 13 Department of Biological Sciences, Virginia Tech, Blacksburg, Virginia USA; 14 Chair of Hydrobiology and Fisheries, Institute of Agricultural and Environmental Sciences, Estonian University of Life Sciences, Tartu, Estonia; 15 Norwegian Institute for Water Research, Økernveien, Norway; 16 University of Massachusetts Lowell, Lowell, USA; 17 Dept of Ecoscience, Aarhus University, Aarhus, Denmark; 18 U.S. Geological Survey, Southwest Biological Science Center, Grand Canyon Monitoring and Research Center, Flagstaff, AZ USA; 19 Department of Aquatic Sciences and Assessment, Swedish University of Agricultural Sciences, Uppsala, Sweden; 20 Israel Oceanographic and Limnological Research, The Yigal Allon Kinneret Limnological Institute, Migdal, Israel; 21 CSIRO, Canberra, Australia; 22 University of Wisconsin–Madison, Madison, USA; 23 Environmental Sciences Division, Oak Ridge National Laboratory, Oak Ridge, TN 37831 USA; 24 Deptartment of Plankton and Microbial Ecology, Leibniz Institute of Freshwater Ecology and Inland Fisheries, Stechlin, Germany; 25 Biochemistry and Biology, Potsdam University, Potsdam, Germany; 26 Australian Rivers Institute, Griffith University, Griffith, Queensland Australia; 27 Department of Ecology and Evolutionary Biology, Cornell University, Ithaca, NY 14853 USA; 28 Faculty of Life and Environmental Sciences, University of Yamanashi, Yamanashi, Japan; 29 Institute for Atmospheric and Earth System Research, University of Helsinki, Helsinki, Finland; 30 Robert B. Annis Water Resources Institute, Grand Valley State University, Allendale, USA; 31 Finnish Meteorological Institute, Helsinki, Finland; 32 Finnish Environment Institute (Syke), Helsinki, Finland; 33 University of Southern Denmark, Biological Institute, Campusvej 55, Odense, 5230 Denmark; 34 Natural Resources Institute Finland (Luke), Helsinki, Finland; 35 Department of Environmental Sciences, University of Stockholm, Stockholm, Sweden; 36 Centre for Northern Studies and Centre Eau Terre Environnement, Institut national de la recherche scientifique, Québec, QC Canada; 37 Department of Environmental Science, University of Basel, Basel, Switzerland; 38 Faculty of Geography, Yunnan Normal University, Kunming, China; 39 Department of Environmental and Biological Sciences, University of Eastern Finland, Kuopio, Finland; 40 Biology Centre CAS, Institute of Hydrobiology, Na Sádkách 7, 370 05 České Budějovice, Czechia; 41 McCord Environmental and University of California Davis, Davis, CA 95616 USA; 42 Department of Ecology and Geology, Marine Ecology and Limnology Research Group, Universidad de Málaga, Málaga, Spain; 43 Department of Chemistry, Life Sciences and Environmental Sustainability, University of Parma, Parma, Italy; 44 Aquatic Physics Group, Department F.-A. Forel for Environmental and Aquatic Sciences (DEFSE), Faculty of Science, University of Geneva, Geneva, Switzerland; 45 Department of Geography and Planning, School of Environmental Sciences, University of Liverpool, Liverpool, United Kingdom; 46 Centre Eau Terre Environnement, Institut national de la recherche scientifique, Québec, Canada; 47 Département des sciences fondamentales, Université du Québec à Chicoutimi, Saguenay, Canada; 48 Karst Research Team, Chongqing Key Laboratory of Carbon Cycle and Carbon Regulation of Mountain Ecosystem, School of Geography and Tourism, Chongqing Normal University, Chongqing, 401331 China; 49 Department of Biology, Aarhus University, CIFAR Research Centre, Ole Worms Allé 1, 8000 Aarhus C, Denmark; 50 Research Center for Limnology and Water Resources, Indonesia National Research and Innovation Agency (BRIN), Jakarta, Indonesia; 51 Eawag, Department of Surface Waters-Research and Management, Kastanienbaum, Switzerland; 52 Limnological Institute, Department of Biology, University of Konstanz, Konstanz, Germany; 53 Reservoir Doctors, Canberra, Australia; 54 Environmental Science Department, Dickinson College, Carlisle, PA 17013 USA; 55 National Institute for Environmental Studies, Ibaraki, Japan; 56 Ecology and Genetics/Limnology, Uppsala University, Uppsala, Sweden

**Keywords:** Limnology, Carbon cycle

## Abstract

Lakes, ponds, and reservoirs (hereafter: “lakes”) are important sources of the greenhouse gases carbon dioxide (CO_2_) and methane (CH_4_). Emissions of CO_2_ and CH_4_ from lakes are regulated in part by in-lake processes, including the production and storage of gases in the lower parts of the water column (bottom waters). However, while substantial efforts have been made to improve estimates of greenhouse gas emissions from lakes, limited data on gas concentrations along depth profiles have prevented the incorporation of bottom-water processes in global emission estimates. Here, we present GHG-depths: the largest existing dataset of depth-profile CO_2_ and CH_4_ measurements worldwide, including 522 lakes across 38 countries and all seven continents. These data include contributions from 45 research teams and 56 published studies, totaling 2558 discrete sampling events. As global change continues to alter biogeochemical cycling in lakes, these data can help improve mechanistic models to better predict greenhouse gas production and emission from lakes worldwide.

## Background & Summary

Lakes, ponds, and reservoirs (hereafter: “lakes”) play an important role in the global carbon cycle, as they actively transform, sequester, and emit carbon to the atmosphere^1,2^. Lakes are globally significant sources of the greenhouse gases carbon dioxide (CO_2_) and methane (CH_4_), releasing an estimated 1.07–2.35 Pg CO_2_^3^ and 37–156 Tg CH_4_^4^ into the atmosphere per year, although the actual magnitude of emission is highly uncertain. Emissions of CO_2_ and CH_4_ are influenced by both external hydrologic inputs^5–8^ and internal biogeochemical processes. In particular, emissions are largely mediated by microbial processes in the lower parts of the water column (bottom waters) and sediment of many lakes, where CO_2_ and CH_4_ can accumulate at high rates due to respiration and methanogenesis^9^. Accumulated gases can then be emitted into the atmosphere via diffusion to surface waters and ebullition from sediment, or be suddenly released during sporadic mixing events, driving both the magnitude and seasonal patterns of gas emissions^9–13^. In reservoirs, this buildup can also contribute to CO_2_ and CH_4_ degassing emissions downstream of the dam site^14^, such as when waters with high CO_2_ and CH_4_ concentrations are sent through hydropower turbines, a process that is estimated to contribute 12 Tg CH_4_ yr^−1^ from reservoirs globally^3,4^. While substantial efforts have been made to improve estimates of lake emissions^15–18^ and surface water dissolved gas concentrations^19,20^, measures of CO_2_ and CH_4_ concentrations across the depth profile remain less common on a global scale, which limits the incorporation of bottom-water processes to inform greenhouse gas production and emissions estimates via global models.

While broad-scale drivers of CO_2_ and CH_4_ concentrations may be similar between surface and bottom waters (e.g., respiration^13^, lake primary productivity^21^, or lake size^16^), spatiotemporal patterns of bottom-water concentrations can fundamentally differ from surface concentrations. In stratified lakes, gas exchange is limited between bottom and surface waters, leading to the buildup of CO_2_ and CH_4_ and the depletion of oxygen in bottom waters^22^. Anoxia can further increase CH_4_ accumulation as anaerobic methanogenesis proceeds and aerobic methanotrophy becomes limited under low oxygen conditions (bottle incubations suggest optimal oxygen for methanotrophy around 15 µmol O_2_ L^−1^)^23^. When stratification breaks down, rapid mixing of the water column can lead to increased emissions of stored gases (storage flux), especially after long periods of stratification, such as in dimictic or monomictic lakes^24–27^. Shallow polymictic systems can also experience periods of gas buildup, with intermittent mixing events leading to pulses of increased emissions^10,11,28^. Ultimately, oxygen dynamics, primary productivity, and the duration of stratification all control CO_2_ and CH_4_ accumulation, which determines the magnitude of storage flux following lake mixing events. As global change is predicted to shift both lake stratification and oxygen regimes (e.g., stronger stratification, more prevalent anoxia, more frequent storms, etc.)^29,30^, bottom-water gas dynamics, and thus emission rates, are expected to be impacted across lakes worldwide.

**Fig. 1:**
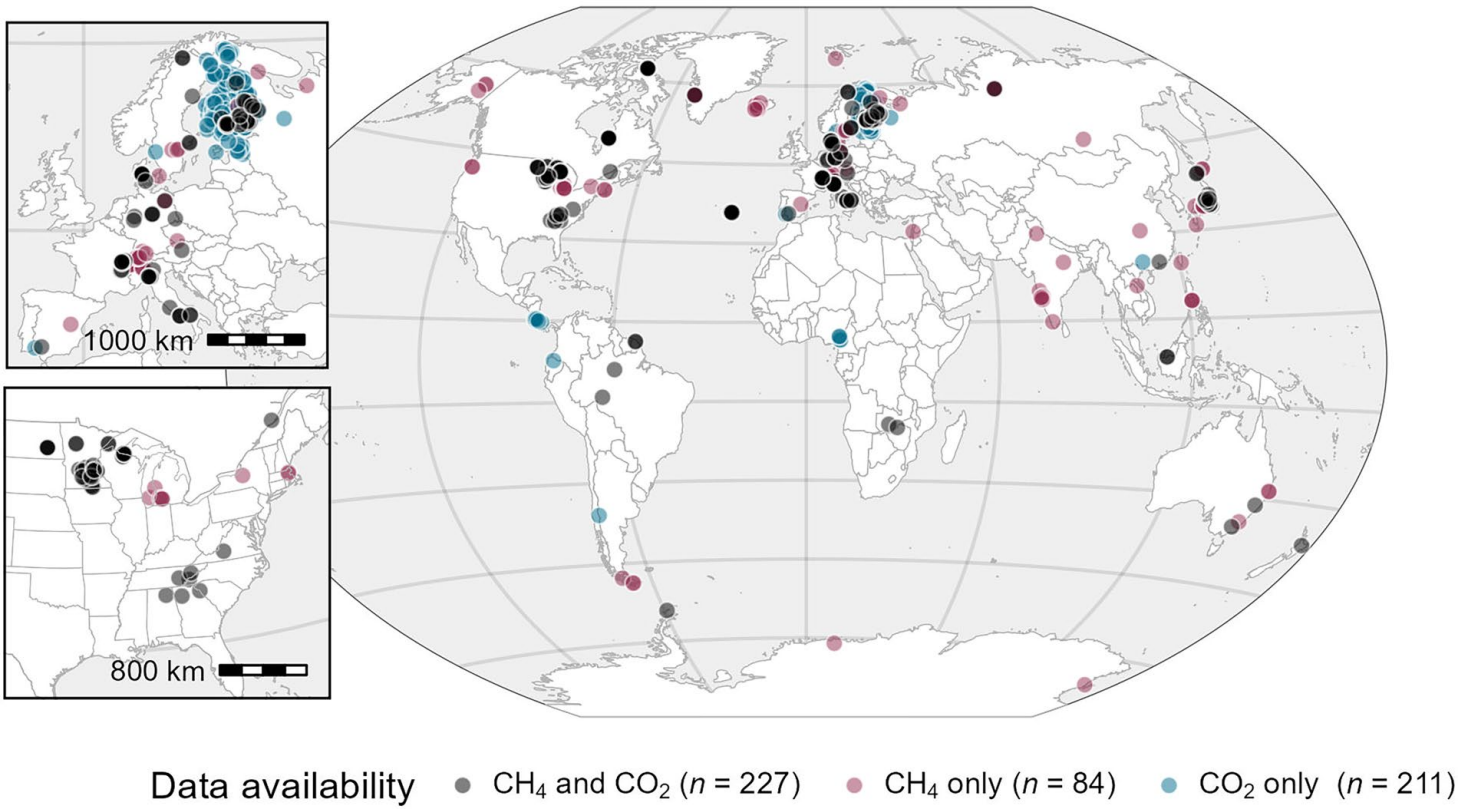
Map showing the locations of the 522 water bodies (lakes, reservoirs, and ponds) included in the dataset, with markers indicating the availability of CH_4_ (red), CO_2_ (blue), or data for both gases (black). The top left inset shows European sites, while the bottom left inset shows sites in the eastern United States. Base map made with Natural Earth^135^.

**Fig. 2:**
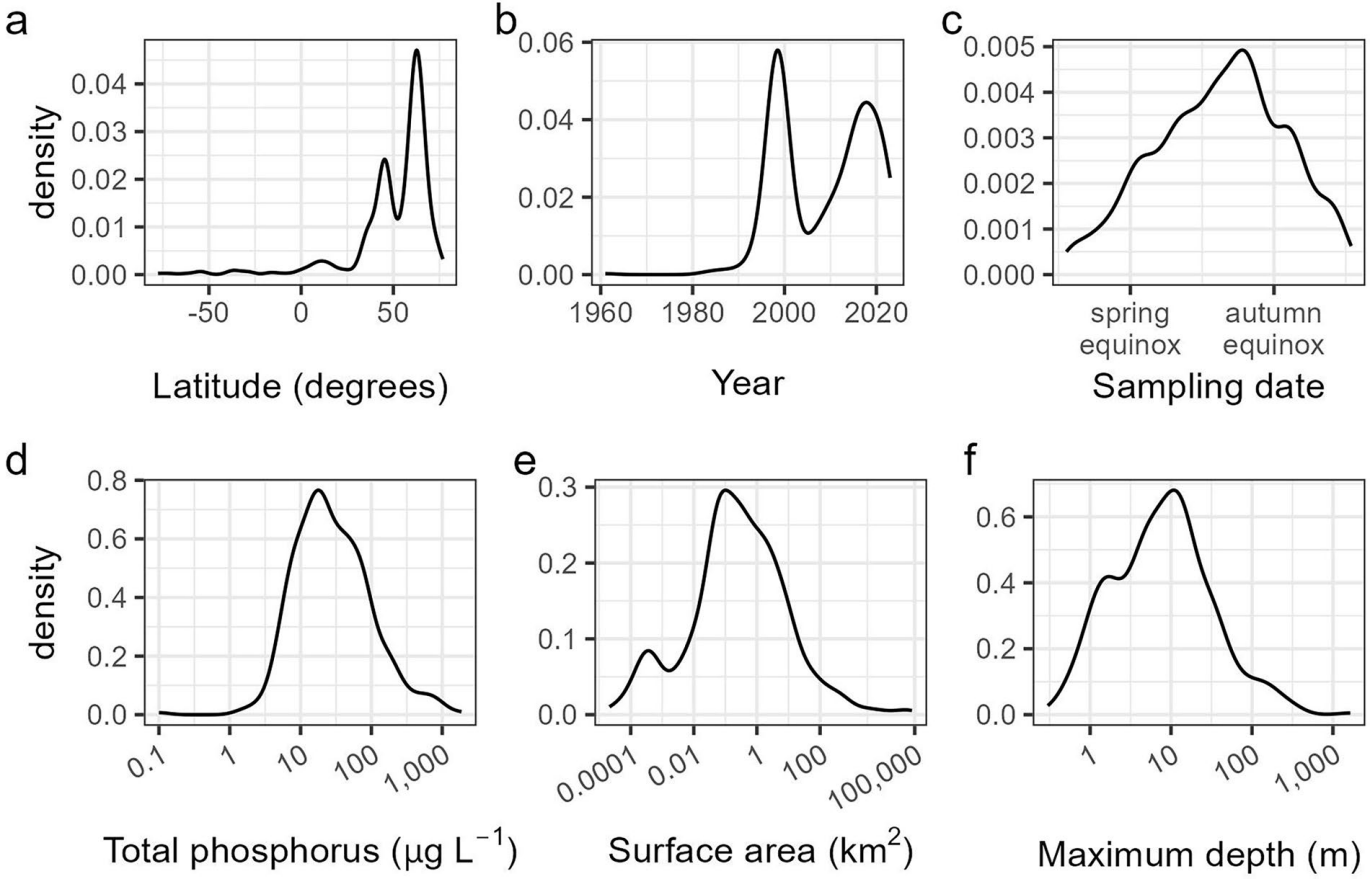
Histograms showing: the range of lake latitudes (**a**), years when data were collected (**b**), seasonal sampling dates across all samples, corrected for hemisphere (**c**), lake mean total phosphorus concentrations (**d**), lake surface area (**e**), and maximum lake depth (**f**). Density represents the relative frequency of data points.

Here, we present GHG-depths: a geographically extensive dataset of depth-profile CO_2_ and CH_4_ concentrations, along with environmental drivers, from 522 lakes^31^. These data span 7 continents and 38 countries, with a total of 2,558 discrete sampling events (Fig. 1). Data from multiple sampling dates are included for 373 lakes, and 87 lakes include data from multiple years. Data include site-level characteristics and lake-specific morphological properties, as well as sample-specific water quality and chemical variables, with an emphasis on depth-resolved temperature (485 lakes) and dissolved oxygen (DO) concentrations (393 lakes). Lakes within the dataset encompass a broad latitudinal gradient, with surface area, depth, and total phosphorus concentrations each spanning multiple orders of magnitude (Fig. 2).

By compiling these data, we aimed to bridge the knowledge gaps between global patterns of surface and bottom-water CO_2_ and CH_4_ dynamics in lakes. The GHG-depths dataset integrates observations from a wide array of climatic regions, lake morphologies, and trophic states, including often underrepresented systems from the global south. This dataset provides a basis for understanding how physical and environmental drivers influence gas accumulation in lakes at both regional and global scales. Additionally, this dataset can be used to inform and calibrate process-based lake models to incorporate bottom-water processes into estimates of lake emissions^32–34^. Ultimately, the utilization of this dataset not only improves the mechanistic understanding of in-lake gas dynamics but can also inform more accurate models for upscaling of global greenhouse gas emissions, which is essential for projecting the trajectory of climate change.

The GHG-depths dataset is released for noncommercial use only and is licensed under a Creative Commons Attribution 4.0 International License (CC BY 4.0). All publications that use GHG-depths are encouraged to appropriately cite the data and this paper, and to contact and collaborate with the site-specific data providers for expertise in interpreting data when appropriate.

## Methods

**Fig. 3:**
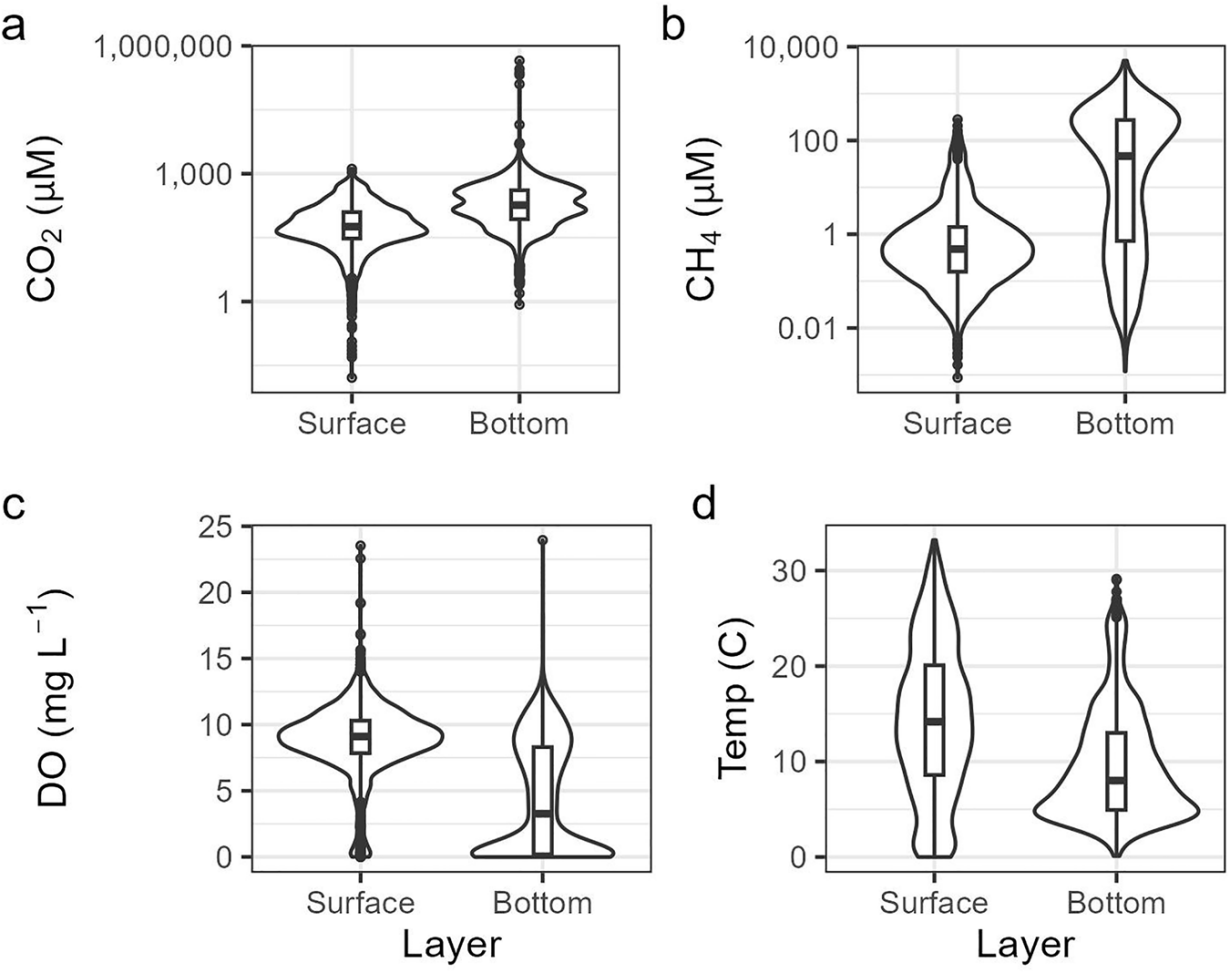
Violin plots showing the distribution of CO_2_ (**a**), CH_4_ (**b**), dissolved oxygen (DO; **c**), and temperature (**d**) in the surface and bottom layer of each unique profile included in the dataset where data was available. The surface layer represents the value at the shallowest depth in a unique profile (must be within the top third of the water column), while the bottom layer represents the value at the deepest depth (must be within the bottom half of the water column). Boxplots within the violin plots represent the first quartile, median, and third quartile, with outliers represented as points.

We compiled depth-profile CO_2_ and CH_4_ concentration data from 522 lakes worldwide (Fig. 1), including 49 lakes from unpublished sources, and 473 lakes from previously published studies^7,10–13,22,25,35–127^ (Table 1). CO_2_ and CH_4_ concentrations in the GHG-depths dataset span seven and six orders of magnitude, respectively, with distinct differences between surface and bottom waters (Fig. 3). This project started as a working group within the Global Lake Ecological Observatory Network (GLEON, https://gleon.org), with the aim of characterizing bottom-water greenhouse gas dynamics in lakes worldwide. We first issued a call for collaboration to GLEON members to contribute data. To broaden the scope of our dataset, we identified additional published data sources using a standardized literature review (detailed methods below) and contacted corresponding authors to acquire original datasets. For the remaining publications (e.g., if we could not reach the author), we manually extracted data directly from the publication.

**Table 1:** Counts (n) of waterbody type and data availability that are included in the GHG-depths dataset.

Waterbody type	Total (n)	CO_2_	CH_4_	Water temperature	Dissolved oxygen	Multiple dates
Lakes	382	320	179	356	275	298
Ponds	90	85	85	83	79	45
Reservoirs	50	35	47	46	39	30
All	522	440	311	485	393	373

Ponds are waterbodies <0.05 km^2^ in surface area and <5 m in maximum depth.

### Inclusion criteria

We employed broad inclusion criteria to maximize lake coverage while ensuring data comparability and usability. To be included in the GHG-depths dataset, contributions required concentration data for CO_2_ or CH_4_ at depth and datasets containing only surface measurements or emissions data were excluded. We requested submissions of depth-profile data with at least two depth-resolved measurements within the water column, and ultimately all but 11 lakes had concentration data from multiple depths (i.e., depth-resolved profiles). We required that measurements were from inland lentic systems (lake, pond, or reservoir) but did not impose any requirement on the stratification regime or stratification status at the time of sampling in that lake. We did not classify data points as belonging to the epilimnion or hypolimnion, though for one lake (Lake Onego, also referred to as Lake Onega) exact depths were not available, and the water column layer is recorded within the “Depth_layer” column. We required that dissolved gases were measured directly (e.g., using headspace equilibration method or sensor) rather than modeled or estimated (e.g., using dissolved inorganic carbon; DIC). We made an exception for Lake Nyos, Cameroon, where dissolved CO_2_ concentrations are extremely high and the relationship between DIC and CO_2_ has been extensively validated^58^. While we made every effort to include complete auxiliary measurements for each lake, we did not exclude lakes due to limited availability of auxiliary data.

### Data submission

We used standardized templates for data contributions, which are included in the data package as “Data submission templates”^31^. Profile data templates included columns for sample information (contact name, lake name, sampling site, date, sample depth), CO_2_ and CH_4_ concentrations, and auxiliary environmental data (dissolved oxygen, temperature, pH, total phosphorus, total nitrogen, and specific conductance). Lake information templates included contact information, lake geography and morphometry (latitude, longitude, maximum depth, mean depth, surface area, volume), CO_2_ and CH_4_ sampling methods (e.g., sensors, headspace equilibration method, etc.), stratification regime, trophic status, lake type (reservoir/lake/pond), lake-specific water chemistry means (chlorophyll *a*, total nitrogen, total phosphorus, dissolved organic carbon, pH), mean residence time, the time period over which chemistry and residence time means were calculated, a reference to any published data product or publication, and any additional relevant notes. To avoid errors associated with unit conversions, we asked that all numerical data be contributed as values in the original units, with units specified separately in adjacent columns. After compiling data, we then algorithmically standardized units for all variables. For all unpublished data, the methods of greenhouse gas data collection are included in Table 2.

**Table 2:** Methods for unpublished data. Lakes with the same methods were grouped together.

Lake Name	Methods
Římov Reservoir	Headspace (N_2_) technique according to McAuliffe 1971^133^; 20 mL vials; dissolved CH_4_ (measured on a gas chromatograph (GC)) concentrations were calculated with the solubility coefficient.
Svartvatnet	Headspace method. 120 mL glass bottles filled with lake water sampled with a Niskin bottle bubble free overflowing ca. 3 times, closed without bubbles with butyl rubber stopper; treated with NaOH. 20 mL N_2_ headspace was introduced in the lab, analysed after mininum 1 hour of equilibration with a GC-flame ionization detector (FID).
Little Long, North Twin	Headspace methods following the method in Cadieux *et al*.^40^.
Magelungen, Djurgårdsbrunnsviken	Headspace method. Samples were equilibrated with ambient air and analysed using a Picarro GasScouter fitted with a sampling loop.
Lunz	Headspace method. Water samples from different depths were extracted in a syringe and filled with a headspace of zero air, shaken for one minute, and headspace sample was measured with a gas analyzer (Picarro G2201-i Isotopic Analyzer).
Fure, Søholm	Headspace method. Water samples were extracted and filled with ambient air, shaken for two minutes, and a headspace sample extracted using a syringe which was measured with a GC (Trace 1300 Gas Chromatograph, Thermo Scientific, Italy, TG-Bond Msieve 5 A flame ionization detector).
Rh1, Rh2, Rh3, Rh4, Rh5, Rh6	Headspace method. Water samples were extracted and filled with ambient air, shaken for two minutes, and a headspace sample extracted using a syringe which was measured with a GC.
Bautzen Reservoir, Möhne Reservoir, Bigge Reservoir	Headspace method. Water samples were extracted in a syringe and filled with a headspace of ambient air, shaken for one minute, and headspace samples stored in Exetainer. Analysis by GC (SRI, with FID and methanizer).
Eagle, Freeborn, Goose, Henry, Hook, Albert Lea, Kasota, McMahon, Oak Leaf, Pickeral, Silver, Swan, Swartout, Union, Cedar (H), Cedar (NP), Clear, Cody	Headspace method. Water samples were extracted in a syringe and filled with a headspace of ambient air, shaken for two minutes, and the headspace sample was measured with a portable gas analyzer (ABB GLA131).
Elk, Itasca	Headspace method. Water samples were extracted in a syringe and filled with a headspace of ambient air, shaken for two minutes, and the headspace sample was measured with a portable gas analyzer (DX4040 FTIR Gas Analyzer, Gasmet Technologies Oy, Vantaa, Finland).
Ikeda, Panketo, Yunoko, Akan	Headspace methods following the method in Magen *et al*.^134^.
Ward	Lake water (45 mL) was extracted from a Van Dorn using a 60 mL syringe and an airtight valve installed on the side of the Van Dorn sampler. Upon returning to the lab (<1 h from sampling), a 15 mL N_2_ gas headspace was added to 45 mL of water in each syringe. The syringes were vigorously shaken and allowed to sit for 30 min to allow for headspace equilibration. The headspace was analyzed for CH_4_ on an Agilent 6890.

For published data, we refer readers to the methods provided in the publications from which the data were sourced (see the data_reference field of “Lake information” for publication citations and the CH4_method and CO2_method fields for brief summary of methods).

### Literature review

To systematically identify publications with relevant data, we conducted a literature review. On 9 February 2023, we searched the Web of Science Core Collection database (Clarivate Analytics, Philadelphia, USA) for “(hypolimn*) and (carbon dioxide or methane or greenhouse gas or CO_2_ or CH_4_),” which yielded 291 publications. We then manually reviewed the full texts of these publications to identify those that included depth-profile measurements of gas concentrations in a lake, pond, or reservoir, following our *Inclusion Criteria*, above. This systematic search process reduced the number of publications to 110. This search was restricted to English-language literature, which limits global representation; future compilations that broaden language inclusion would likely yield a more global set of lakes. Whenever possible, we preferred to include the authors on the team of collaborators rather than extracting data, recognizing the effort required for data collection and the value of local expertise in interpreting data from individual lakes. We reached out to the corresponding author of each publication over email to invite them to contribute data (following *Data submission* methods above). If the author declined or did not respond after two follow-up emails, our team then manually extracted data from the publication.

We extracted data and lake information from the figures, tables, or supplementary information of the publication using the same data templates described above (*Data submission*). If profile data were only presented in figures, we used the tool WebPlotDigitizer (https://automeris.io) to extract data from each figure. When necessary, we filled in missing lake information using other published literature from the same lake. In total, we extracted data from 56 publications^7,12,25,75–127^.

### Additional lake data

We augmented our compiled dataset with additional lake information to expand the utility of these data for future studies. While discrete lake classifications (e.g., stratification regime and trophic state) do not describe all of the environmental variability across lakes^128^, these classifications can be used for comparing across systems when more precise data are not available. If no contributor-reported trophic state was available, we classified trophic state using available chlorophyll *a* and/or total phosphorus data^129^. For lake type, we classified systems using lake surface area and maximum depth thresholds into ponds (<0.05 km^2^ surface area; <5 m maximum depth), small lakes (<0.05 km^2^; >5 m), shallow lakes (>0.05 km^2^; <5 m), and lakes (>0.05 km^2^; >5 m)^130^. Reservoirs were retained as a separate lake type when reported by contributors. For lakes with multiple sample sites, the deepest site was identified as the focal site for comparisons across lakes.

To broaden the interoperability of our compiled dataset, we matched the lakes in our dataset with their unique lake IDs from HydroLAKES, a global database of 1.4 million lakes with a surface area ≥ 0.1 km^2^ ^131^. HydroLAKES IDs have been incorporated in multiple published lake data products, creating a common reference system that allows future studies to match the CO_2_ and CH_4_ concentration data compiled here with additional data. We algorithmically overlaid lake polygons from HydroLAKES with the latitude and longitude of lakes in our dataset to identify which HydroLAKES ID corresponded to each lake, and we manually ensured that lake names and surface area corresponded between both data products. In total, 237 of the lakes in this dataset had matching HydroLAKES IDs.

### Analysis-ready compiled greenhouse gas data file

To aid in future research, we generated an analysis-ready greenhouse gas data file that harmonizes lake temperature, dissolved oxygen, and greenhouse gas measurements for each sampling date at each lake. In data extracted from publication figures, measurement depths may differ slightly between water temperature, dissolved oxygen, and greenhouse gas data, limiting the ability to directly assess drivers of dissolved gas concentration. Consequently, in the analysis-ready data product, we linearly interpolated and averaged all temperature and dissolved oxygen data from published article figures to 0.1 m intervals (sample depth ≤10 m) or 1 m intervals (sample depth >10 m) and matched these values with measured CO_2_ and CH_4_ concentration data. We restricted this datafile to observations containing greenhouse gas measurements and included only a single focal site for each lake, typically the deepest point, excluding additional sites when multiple were available.

## Data Records

**Fig. 4:**
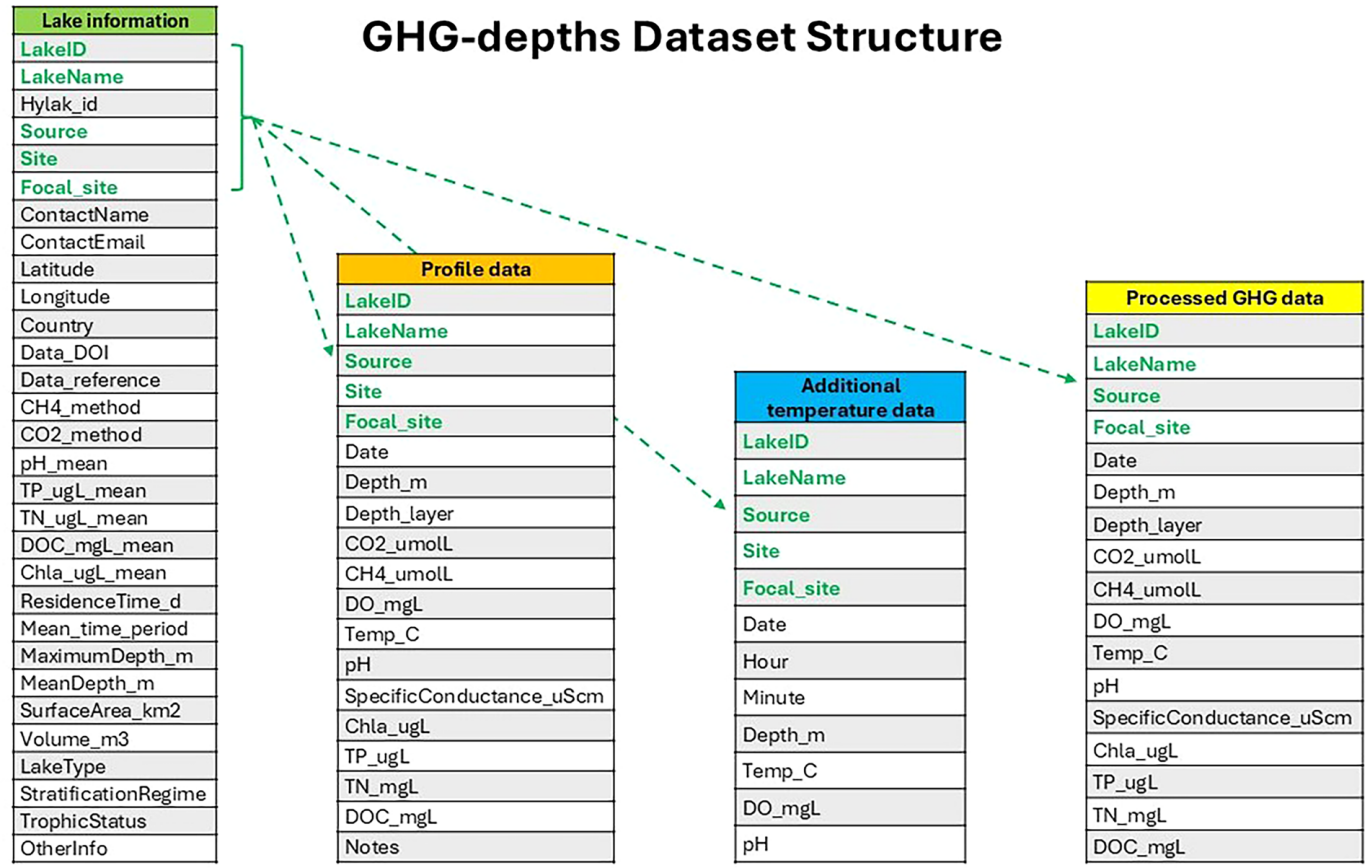
Structure of the files included in the GHG-depths dataset. Column names and arrows shown in green represent fields that can be used to join the Lake information file with the remaining data files.

The GHG-depths data publication includes four files, which are linked via unique lake IDs (LakeID column), sampling site (for lakes with multiple sampling sites; Site column), and data sources (Source column; Fig. 4). “Lake information” presents lake-level or site-level data and metadata for all sites, including contributor information, lake characteristics, methods, and additional information. “Profile data” presents all compiled depth-profile data, including greenhouse gas concentrations and auxiliary measurements (e.g., temperature, dissolved oxygen, etc). “Additional temperature data” provides supplemental temperature data, including temporally resolved sensor data that may be used for assessing stratification dynamics in each lake. Finally, “Processed GHG data” includes analysis-ready CO_2_ and CH_4_ data, with the corresponding interpolated temperature and dissolved oxygen data, as described above (*Analysis-ready compiled greenhouse gas data file*). Note that this “Processed GHG data” file contains only data from the focal site at each lake (typically the deepest point), whereas the “Lake information” file may include additional sites for some lakes. Therefore, the “Focal_site” field must be used when joining lake information to the processed GHG file, to make sure site-level data from the correct site is used (Fig. 4). All files are csv files available at the Environmental Data Initiative, accessible at 10.6073/pasta/2b72b89bbfbb3da0e198f392a9cbad18^31^.

## Technical Validation

We assessed the quality of the data included in the GHG-depths dataset through multiple steps to address any errors that may have occurred during the compilation and harmonization of disparate data sources. After initially compiling the data, we visually inspected gas concentrations and environmental driver data using histograms and plots to identify outliers. Obvious mistakes and discrepancies were corrected, and any problematic data were removed from the dataset. Next, we generated summary reports for all collaborators to review, including contributors of unpublished data and article authors. Reports summarized and visualized each collaborator’s data within the combined dataset, in addition to displaying raw data and metadata for each individual sampling event. Collaborators reviewed these reports and provided feedback on any remaining issues (e.g., inconsistencies in date formats or incorrectly entered geographic coordinates). In cases where data were extracted directly from papers, similar reports were reviewed by the same person who collected the data to ensure that the data were complete and correct. Overall, we found that the process of generating these reports and receiving feedback dramatically improved the reliability of the dataset and leveraged our large contributor team.

## Data Availability

Data are available in the Environmental Data Initiative repository^31^, and accessible at 10.6073/pasta/2b72b89bbfbb3da0e198f392a9cbad18.

## Code availability

All data compilation and figure generation was conducted using R statistical software^132^. Code used to generate figures, validate data, and generate the analysis-ready compiled greenhouse gas data file (“Processed GHG data”) is available in an open-source GitHub repository (10.5281/zenodo.17782010).
